# Draft genomes of *Blastocystis* subtypes from human samples of Colombia

**DOI:** 10.1186/s13071-022-05619-7

**Published:** 2023-02-02

**Authors:** Adriana Higuera, Dayana E. Salas-Leiva, Bruce Curtis, Luz H. Patiño, Dandan Zhao, Jon Jerlström-Hultqvist, Marlena Dlutek, Marina Muñoz, Andrew J. Roger, Juan David Ramírez

**Affiliations:** 1 Centro de Investigaciones en Microbiología y Biotecnología-UR (CIMBIUR), Facultad de Ciencias Naturales, Universidad del Rosario, Bogotá, Colombia; 2 Department of Biochemistry, University of Cambridge, Hopkins Building, Downing Site, Tennis Court Road, Cambridge, CB2 1QW United Kingdom; 3 Institute for Comparative Genomics (ICG), Department of Biochemistry and Molecular Biology, Dalhousie University, Halifax, NS B3H 4R2 Canada; 4 Department of Cell and Molecular Biology, BMC, Uppsala Universitet, Box 596, 751 24 Uppsala, Sweden; 5 Molecular Microbiology Laboratory, Department of Pathology, Molecular and Cell-Based Medicine, Icahn School of Medicine at Mount Sinai, New York City, NY USA

**Keywords:** *Blastocystis*, Comparative genomics, Genomic diversity, Nanopore sequencing

## Abstract

**Background:**

*Blastocystis* is one of the most common eukaryotic microorganisms colonizing the intestines of both humans and animals, but the conditions under which it may be a pathogen are unclear.

**Methods:**

To study the genomic characteristics of circulating subtypes (ST) in Colombia, we established nine xenic cultures from *Blastocystis* isolated from human fecal samples, we identified 10 different subtypes, since one sample had a mixed infection. Thus, the genomes of the subtypes ST1 (*n* = 3), ST2 (*n* = 1), ST3 (*n* = 2), ST6 (*n* = 1), ST7 (*n* = 1), and ST8 (*n* = 2) were sequenced using Illumina and Oxford Nanopore Technologies (ONT).

**Results:**

Analyses of these draft nuclear genomes indicated remarkable diversity in terms of genome size and guanine-cytosine (GC) content among the compared STs. Illumina sequencing-only draft genomes contained 824 to 2077 scaffolds, with total genome size ranging from 12 to 13.2 Mb and N_50_ values ranging from 10,585 to 29,404 base pairs (bp). The genome of one ST1 isolate was sequenced using ONT. This assembly was more contiguous, with a size of 20 million base pairs (Mb) spread over 116 scaffolds, and an N_50_ of 248,997 bp.

**Conclusion:**

This work represents one of the few large-scale comparative genomic analyses of *Blastocystis* isolates, providing an additional glimpse into its genomic diversity.

**Graphical Abstract:**

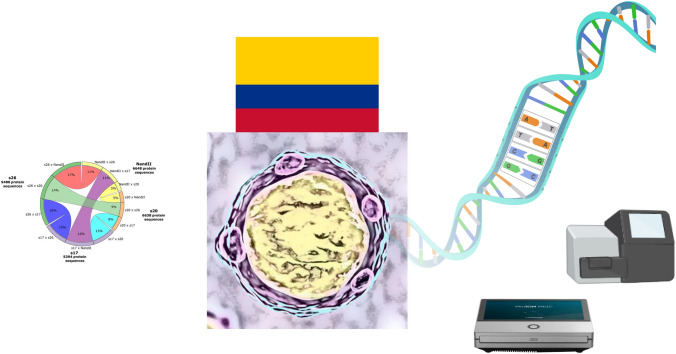

**Supplementary Information:**

The online version contains supplementary material available at 10.1186/s13071-022-05619-7.

## Background

*Blastocystis* is a ubiquitous anaerobic protist, mainly found in the large intestine of a wide range of animals and humans. It has been reported either as a constituent of healthy microbiota [1, 2] or associated with intestinal dysbiosis, although this is still debated [3, 4]. Most knowledge of genetic diversity amongst *Blastocystis* isolates comes from analyses of the small subunit ribosomal gene (18S ribosomal DNA [rDNA]), which has allowed the detection of different subtypes (STs) associated with various hosts [5–8]. Sequencing of 18S rDNA amplicons [9] is the standard method for the identification of STs which may be present in mixed infections with varied population densities [6, 10]. *Blastocystis* 18S rDNA sequences obtained from a variety of different hosts and from different global regions group into at least 30 STs [11, 12]. Recently, an Oxford Nanopore Technologies (ONT) platform was implemented to obtain the complete sequence of the 18S rDNA from cultures and stools, allowing the validation of new STs [13].

Despite the high diversity of *Blastocystis* STs discovered so far, there have been very few genomic studies; only three high-quality genomes have been published to date. The first genome reported was that of ST7, which was Sanger-sequenced from a fecal sample of a symptomatic human [14]. Then, the Illumina-only assembly of the ST4 genome, also from a human fecal sample, was published [15], followed by a hybrid 454 pyrosequencing/Illumina assembly of ST1 obtained from the American Type Culture Collection (ATCC 50177) [16]. Genome assemblies have been reported for ST2, ST3, ST4, ST6, ST8, and ST9 (only six of the 30 STs known so far) [1], but these lack gene predictions (only RNA sequencing [RNA-Seq] data for ST1). Gene prediction in *Blastocystis* is inherently difficult in part because of the lack of genome-encoded stop codons in a significant fraction of genes. These genes have stop codons that are created post-transcriptionally by polyadenylation, a process that relies on a conserved sequence motif downstream of the polyadenylation site [16].

Given the lack of sufficient genomic data for *Blastocystis*, a reference and well-assembled genome, and genomes from the Americas coupled with the imperative need for new methodologies to study its genomic diversity, we report herein the sequencing, assembly, and analysis of nuclear genomes of *Blastocystis* ST1, ST2, ST3, ST6, ST7, and ST8 isolated from human feces from Colombia. First, we present a comparison between the genomes assembled from the different STs obtained in this study and those previously published for ST1, ST4, and ST7. Subsequently, we focus on the ST1 samples generated with both Illumina and ONT data, and compare them with the highly curated publicly available reference genome, with a focus on the presence and absence of genes. Our study provides valuable information on the genomic characteristics of the strains analyzed in Colombia and shows a few characteristics of the architecture of *Blastocystis* ST1.

## Methods

### Culture and DNA/RNA extraction

As part of a previous study published by our group [17], we collected 50 samples from Bogota, Colombia. The samples were assessed for the presence of *Blastocystis* using microscopy. All samples positive for *Blastocystis* via microscopic observation (17 samples) were cultured in Jones medium within 24 h of their collection. Cultivation was carried out under xenic conditions following recommendations by Clark and Stensvold [18]. At the end, only nine samples showed growth in Jones medium; the nine subjects from which the isolates were obtained were under the age of 15 years and had no gastrointestinal symptoms.

From a 10-mL grown culture, *Blastocystis* cells were harvested by centrifugation at 280×*g* for 5 min at room temperature. The supernatant was discarded and the pellet was resuspended in 1 mL of sterile phosphate-buffered saline (PBS). This suspension was carefully placed in 9 mL of Histopaque-1077^®^ (Sigma-Aldrich) and was centrifuged at 2000×*g* for 20 min at room temperature, as some part of the cultured cells were lost during the cleaning with Histopaque-1077^®^ (Sigma-Aldrich) due to the need to separate bacteria from *Blastocystis*. *Blastocystis* cells were collected from the interface and transferred into a clean and sterile tube. For downstream processing, we only considered interface samples that seemed clean (no presence of bacteria) under microscopic visualization. The recovered cells were resuspended in 10 mL of sterile 1× PBS and were centrifuged at 500×*g* for 5 min. The pellet containing the protist was retrieved for DNA extraction. Two different methodologies were implemented for DNA extraction according to the subsequent sequencing method. All samples were sequenced with Illumina. For this, around 10 tubes of 10 mL per strain were cultured, and several pellets from the same *Blastocystis* culture were pooled and resuspended in the lysis buffer of the UltraClean Tissue & Cells DNA isolation kit (MoBio-12334), following the manufacturer’s recommendations. Therefore, several tubes were needed to conduct DNA extraction with good concentration and quality for genome sequencing. The final DNA elution was performed on a final volume of 100 µL. After obtaining the DNA, subtyping was conducted as reported elsewhere [17].

Concentrations were higher than 20 ng/µl and quality ratios ranged from 1.8 to 2.0 (260/280) and 2.0 to 2.2 (260/230). Only sample s20 (ST1) was additionally sequenced using ONT. For this, the harvested cells were incubated in 1 mL of Graham lysis buffer (0.1 M EDTA, 0.25% SDS), 4 µL of RNase A (10 U/µL), and 20 µL of proteinase K (10 mg/mL) for 1.5 h at 55 °C. Then, NaCl and cetrimonium bromide (CTAB) were added to the lysis tube to attain concentrations of 0.7 M and 1%, respectively. This mixture was incubated for 15 min at 65 °C. Then, one volume 1× of UltraPure™ Phenol:Chloroform:Isoamyl Alcohol (Invitrogen, Cat No. 15593031) was added, stirred, and centrifuged at 14,000×*g* for 10 min at room temperature. The top aqueous layer was collected in a new tube and the previous step was repeated. Then, the upper aqueous phase was collected, mixed with one volume of chloroform (Sigma-Aldrich, Cat No. 288306), and centrifuged at 14,000×*g* for 10 min at room temperature. Subsequently, the aqueous phase was retrieved and mixed with one volume of isopropyl alcohol at 0.7× (Sigma-Aldrich, Cat No. I9516) and incubated at room temperature for 10 min. This was centrifuged at maximum speed for 20 min at room temperature, and the DNA pellet was washed with 700 µL of 70% ethanol. Finally, the sample was centrifuged at 14,000×*g* for 5 min, the supernatant was removed, and the pellet was resuspended in TE buffer previously heated to 60 °C. The sample was stored at 4 °C overnight, and after that it was stored at −20 °C. The process above was conducted multiple times for sample s20 to obtain enough DNA yield. Hence, all the obtained extractions were pooled, purified with Genomic Tip 20G Kit (Qiagen, Cat No. 10223), and resuspended in 20 µL of TE buffer. The final DNA quantification of sample s20 was 2 µg, with a 260/280 quality of 1.806 and 260/230 of 2.063. Sample s20 was also cultured for RNA extraction using TRIzol (Invitrogen, USA) following the manufacturer’s instructions.

### Sequencing methods

All nine extracted and purified DNA samples were sequenced by Illumina technology on the HiSeq 2000 (150 × 2 paired-end) at the Novogene facility (https://en.novogene.com). Illumina RNA-Seq was only obtained for sample s20 at GenomeQuébec (https://www.genomequebec.com). In addition, sample s20 was sequenced by ONT at Dalhousie University. For this, the library was prepared using native barcoding genomic DNA (SQK-LSK109 with EXP-NBD104) protocol NBE_9065_v109_revL_14Aug2019. The final library (310 ng) was loaded on an R9.4.1 flow cell (no. FAL26917) and sequenced over 72 h on the MinION Mk1B nanopore sequencer.

### Assembly and gene prediction

Illumina reads were quality-trimmed using Trimmomatic [19] with the following parameters: Headcrop: 10, Leading:10, Trailing: 10, Slidingwindow: 40:25, Minlen: 60. The following process was carried out for all samples except sample s20: Trimmed reads were classified into taxonomic groups for Bacteria, Archaea, Eukarya, viruses, and unknown using Centrifuge v1.0.1 [20] and PLAST v2.3.1 [21]. Of these, reads classified as Eukarya and unknown were used to generate an initial assembly with Spades v3.14.1 in metaSPAdes mode [22, 23], and the resulting contigs were again classified using Centrifuge and PLAST against the Nucleotide (nt) database for decontamination purposes. Trimmed reads were mapped on the preliminary decontaminated assembly using Bowtie 2 [24] and retrieved to maximize eukaryotic read availability for obtaining a new and more contiguous assembly using Spades in metaSpades mode. This yielded a nuclear assembly that was manually decontaminated using sequence composition, read depth, and taxonomy information with the assistance of Anvi'o v4.6 [25]. Note that contigs that still lacked taxonomic assignment were additionally evaluated using BLASTN [26] against published *Blastocystis* draft genomes from different STs in NCBI to determine whether they should be kept or discarded. In the case of the sample s8, we found an initial assembly twice the expected size, suggesting a mixed infection; then, the contigs of the assembly were separated into clusters based on the results of the MyCC tool [27], which gives a coordinate for each contig based on the calculation of the depth of coverage, protein search, and kmer usage. In this case, we ran it three times with three modes (4-mer, 5-mer, and 5- and 6-mers). After that, we checked the contigs of each cluster using the hits obtained with Centrifuge, PLAST [21], Diamond [28], and BLASTN with each ST of *Blastocystis* public genomes. Subtyping was confirmed from the raw reads.

Since sample s20 was sequenced with both Illumina and ONT, we first processed the long reads, obtained a preliminary assembly, carried out decontamination, and reassembled selected long reads; then we used the short genomic Illumina reads for error correction and the RNA reads for assisting in gene prediction. In brief, long reads were demultiplexed with deepbinner [29], base-called with Guppy v3.2.4, trimmed with Porechop v0.2.3 (www.github.com/rrwick/Porechop), and filtered by length with Filtlong (https://github.com/rrwick/Filtlong), with minimum mean quality of 10 and minimum length of 1000. The preliminary assembly was performed with Canu v1.9 [30] and all the available long reads. Several decontamination rounds were performed first with Centrifuge and PLAST to separate contigs of Eukaryota or unknown taxonomic affiliation. These were used as templates to recruit long reads using ngmlr (https://github.com/philres/ngmlr), which were used in a reassembly to minimize assembly errors caused by differential read depth coverage when cultures are not axenic. The resulting new nuclear assembly was manually inspected and decontaminated using Anvi'o as described above. The assembly was first polished with Racon [31] and Medaka (https://github.com/nanoporetech/medaka), and error-corrected using the submodule unicycler_polish in Unicycler [32]. Redundant contigs and/or haplotigs were identified and removed with Purge Haplotigs [33]. Once all the genomes were cleaned, assembly statistics were obtained with QUAST [34], and their completion was evaluated with the universal single-copy orthologs—BUSCO v3.0.2 [35] in “genome mode,” with the Eukaryota database with 255 proteins (https://busco.ezlab.org/). Note that the BUSCO analysis was also performed for the publicly available genomes with the same program and database version reported here. GeneMark [36] and BRAKER [37] were used to obtain gene predictions only for s20 genes. RNA-Seq obtained for s20 was also used to predict genes from other ST1 samples (s17 and s26). Predicted gene sets for s17, s20, s26, and NandII were compared against each other using BLASTP [26]. Genes were considered to be present in the other half of the pair if the match had an e-value lower than 1e-30 and greater than 50% coverage of the query.

### Single-nucleotide polymorphism (SNP) analysis

Reads of each genome were mapped to the genome generated with ONT in this study (s20) using Bowtie 2 [24]. The Picard program (v1.85) (http://broadinstitute.github.io/picard/) was used for merging and sorting bam files and marking duplicated reads, as described previously [38]. The SNPs were called among all the samples simultaneously with the population-based UnifiedGenotyper method in the Genome Analysis Toolkit (GATK version 3.4; https://software.broadinstitute.org/gatk/). Later, we realigned and retrieved only the SNPs. GATK VariantFiltration was used to filter low-quality SNPs, according to the following criteria: QD [QualByDepth] < 2.0 ‖ MQ [MappingQuality] < 40 ‖ FS [FisherStrand] > 60.0 ‖ ReadPosRankSum <  −8.0. Finally, the SnpEff program [39] was used to classify all SNPs based on their functional impact. The SNPs obtained were exported into a matrix to perform a comparative analysis, selecting those with a greater number of SNPs classified as high and moderate impact.

## Results and discussion

### Genome assembly of *Blastocystis* STs

**Fig. 1:**
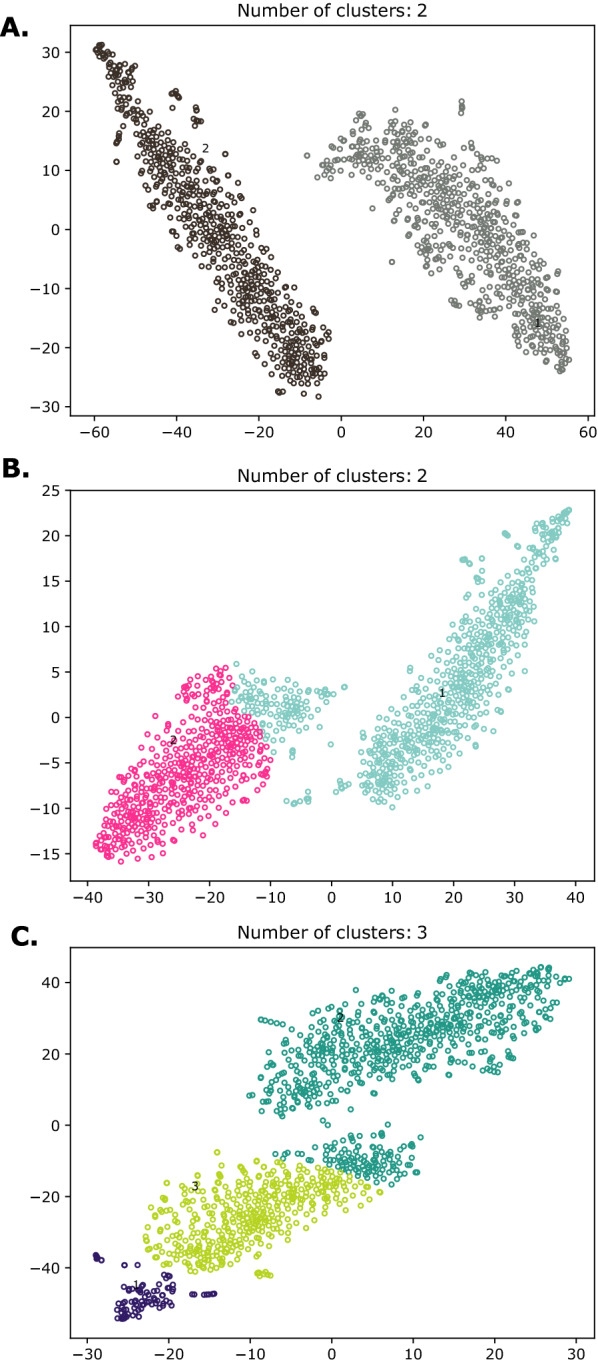
Mixed infection of ST3 and ST6 in sample s8. Clusters obtained with MyCC tool. MyCC gives a coordinate for each contig, based on the calculation of depth of coverage, kmer usage, and protein search: **A** 4-mer, **B** 5-mer, and **C** 5- and 6-mers. Each color represents one cluster which was assigned to ST3 (left cluster) or ST6 (right cluster) using other tools including Centrifuge, PLAST, Diamond, and BLAST

A total of nine isolates were successfully maintained in culture for subsequent DNA extraction and sequencing. These isolates were labeled as s5, s8, s17, s20, s21, s24, s25, s26, and s25 (Table 1). Six different STs were determined following the protocol recommended in previous studies [17]. Interestingly, in the sample s8 we found a mixed infection with ST3 and ST6, which were designated as s8a and s8b, respectively (Table 1; Fig. 1), and we found an initial assembly twice the expected size, suggesting a mixed infection; then, the contigs of the assembly were separated into clusters based on the results of the MyCC tool [27], which gives a coordinate for each contig, based on the calculation of depth of coverage, protein search, and kmer usage. In this case, we ran it three times with three modes (4-mer, 5-mer, and 5- and 6-mers). After that, we checked the contigs of each cluster using the hits obtained with Centrifuge, PLAST [21], Diamond [28], and BLASTN with each ST of *Blastocystis* public genomes, after which the raw reads of each cluster were used to determine the different STs as a suggestive clue of mixed infection.

**Table 1:** Genome statistics for different *Blastocystis* isolates collected in Bogotá, Colombia, as well as published genomes

Genomic features	s20	s17	s26	s5	s21	s8a	S8b	s28	s24	s25	NandII^a^	WR1^a^	Singapore isolate-B^a^
Subtype	ST1	ST1	ST1	ST2	ST3	ST3	ST6	ST7	ST8	ST8	ST1	ST4	ST7
Genome assembly size (Mb)	20.1	12.5	12.5	13.2	12.0	12.1	12.0	12.3	12.7	12.7	16.5	12.9	18.8
Scaffolds	116	1637	2077	1245	896	824	962	852	973	956	580	1,301	54
GC content (%)	54.9	55.0	54.8	54.0	52.1	52.0	43.4	47.1	39.7	39.7	54.6	39.6	45.2
N_50_	248,997	15,245	10,585	21,156	25,307	29,404	27,961	25,077	28,940	28,925	36,659	29,524	296,810

^a^Data for published genomes obtained from https://www.ncbi.nlm.nih.gov/. GenBank assembly accession for NandII GCA_001651215.1, WR1 GCA_000743755.1, and Singapore isolate-B GCA_000151665.1

The assemblies using only Illumina reads had coverage in the range of 30–119× and guanine-cytosine content (GC%) of 39.7–55 (Table 1). Some GC% are similar to those previously reported [16]. As expected, the assembly obtained for s20 is the most contiguous, with only 116 contigs with deep read coverage (average coverage of 1431× with short reads and 74× with long reads). These results motivated us to select s20 as a reference genome compared to NandIII, although only higher-resolution sequencing methods such as Hi-C PacBio will tell us in the future whether this decision was right, and we note this as a limitation of our study. Most assembled genomes were ~12–13 million base pairs (Mb) in size, although ST1 (s20) was the largest (i.e., 20.1 Mb). These sizes may reflect real genome diversity but could also be explained as assembly artifacts; however, we carefully tried to minimize bioinformatics artifacts by performing several assembly and curation rounds. Another explanation might be the differences in DNA concentration or poor control of loading concentration during library preparation when conducting the sequencing that could have affected the final sizes or ultimately assembling using only short reads (Illumina) versus strategies coupling short and long reads. Here, we highlight a limitation of our study, as we were only able to carry out hybrid assembly for s20. Still, future studies should consider assembling *Blastocystis* genomes with short and long reads, or performing more sophisticated sequencing such as Hi-C PacBio in order to obtain long reads that can even help in obtaining chromosome-level assembly as successfully reported for other complex eukaryotic pathogens [40].

**Fig. 2:**
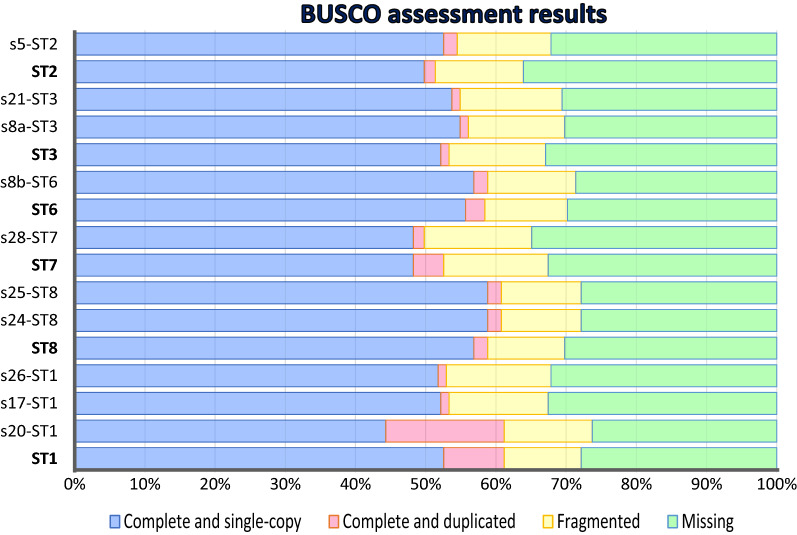
BUSCO assessment. Comparison between complete, fragmented, and missing genes found for each isolate and the published genomes. The subtypes are shown on the *y*-axis, and the percentage of each item determined for BUSCO is shown on the *x*-axis. The STs corresponding to public genomes are in bold

Interestingly, the genome size of the s20 isolate (ST1) was larger than the previously reported ST1 NandII strain [16]. The BUSCO analysis for genome completeness evaluation (Fig. 2) indicated similar levels of completeness among assemblies using the “genome mode” from the same publicly available subtypes. This included s20 when compared to the ST1 (NandII strain), with completeness of 61.2%, including a total of 44.3% (113) complete single-copy and 16.9% (43) complete duplicate genes on a maximum of a set of 255 proteins, while s17 and s26, also from ST1, showed 53.4 and 53% completeness. Of all the BUSCOs evaluated, ST7 had the lowest percentage of complete genes, approximately 49%. These values were estimated using the “genomic mode” because not all our assemblies were used to generate gene predictions. As a result, these estimations are considerably lower than those previously reported for the protein mode by Gentekaki et al. [16]. Note that when using the protein mode in our new ST1 predictions (s20, s17, and s26), the BUSCO completion percentages were 62%, 58.1%, and 56.1%, respectively, which are relatively close to the 67% of ST1 NandII.

**Fig. 3:**
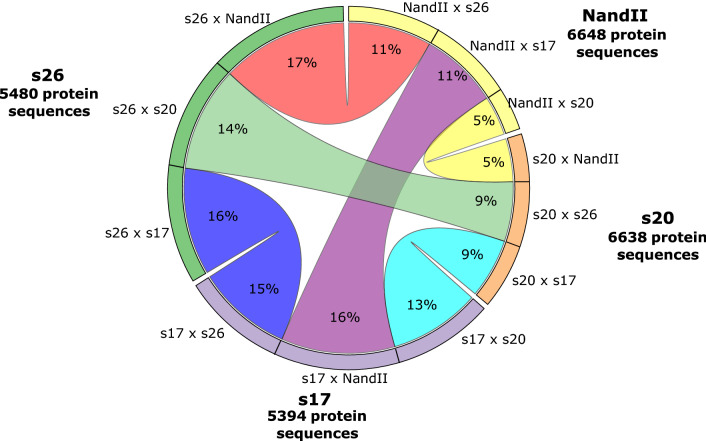
Circos table depicting unique genes between pairs of ST1 (s17, s20, S26) and strain NandII. The percentage of protein-coding gene sets is shown. This represents unique genes when compared in pairs with other STs and vice versa. This comparison is based on BLASTP results with an expected e-value lower than 1e-30 and greater than 50% coverage of the query. In the case of s17× NandII, 16% of genes are unique for s17 in comparison with NandII, and in the case of NandII× s17, 11% are unique for NandII in comparison with s17. The total number of protein sequences is shown for each ST

The predicted gene sets for the ST1 samples (s17, s20, s26) were compared with each other as well as with the published ST1 gene set to determine which genes were unique between pairs (Fig. 3). The percentage of missing genes when genomic gene sets were compared varied from a low of 5% for NandII × s20 and s20 × NandII to a high of 17% when the genes from s26 were compared to the NandII gene set.

In the pairwise comparisons, the percentages are higher when s17 or s26 is compared to NandII or s20 rather than the reverse, mainly because NandII and s20 assemblies have over ~1000 more predicted genes, as shown in Fig. 3, where the number of protein sequences is higher for s20 and NandII. Nevertheless, the differences between the gene sets illustrate the level of genomic diversity among the ST1 samples.

### SNP analysis

The analysis of SNPs was restricted to the samples subtyped as ST1, as the majority of isolates belonged to this ST, including the capacity for a hybrid assembly (s20). The total number of SNPs was 141,835 for isolate s17, 159,677 for strain NandII, and 160,689 for isolate s26 (Additional file 1: Tables S2–S4). The functional impact was classified for each SNP as high (disruptive impact in the protein, like truncation or loss of function), moderate (a non-disruptive variant that might change protein effectiveness), low (harmless or unlikely to change protein behavior), or modifier (usually non-coding variants or variants affecting non-coding genes). A total of 793 SNPs with high impact (Additional file 1: Table S5), 47,910 with moderate impact, 138,315 with low impact, and 97,993 with modifier impact were found. Unfortunately, the genome assembly does not contain whole chromosomes, which is an impediment in analyzing the location of each SNP and constitutes a dramatic limitation of our analysis, as we were not able to estimate ploidy and we could not disregard the fact that the observed SNPs were not the result of ploidy changes. However, we were able to obtain the number of SNPs with high and moderate impact, finding 5265 genes that would be impacted. Those with more than 100 SNPs are shown in Table 2. The gene with the greatest number of SNPs may play a role in the regulation of calcium sequestration, while other genes were related to vesicular protein sorting, cell adhesion, transamidases, or simply hypothetical proteins (Table 2). We detected large numbers of SNPs on each contig, and many coding genes seemed to be impacted by SNPs. These vast changes across SNP density in ST1 samples have been previously described using conserved molecular markers such as 18S and the succinate dehydrogenase gene in Colombia [41, 42]; therefore, our findings seem logical when analyzing the complete genome of these samples. Nevertheless, a chromosome-level assembly is still needed to fully depict the true functional impact of these SNPs, and to start interrogating the multiple consequences of this variation.

**Table 2:** Number of SNPs with high and moderate impact using s20 as a reference genome

Number of SNPs	Product description
388	Sarcoplasmic reticulum histidine-rich calcium-binding protein precursor
350	Hypothetical protein
149	Hypothetical protein, partial
139	Vacuolar protein sorting-associated protein
133	Trichohyalin
130	Protocadherin fat-like protein
126	Protocadherin Fat-like protein
125	Vacuolar protein sorting-associated protein
122	Hypothetical protein
109	Midasin-like protein
105	He PIG protein family

Because interest in *Blastocystis* has increased, the number of subtypes reported has risen as more hosts and geographical regions are sampled, demonstrating the diversity of this microorganism and its versatility depending on the environment in which it is found. Our findings agree with the great variation previously described and demonstrate that, at the genome level as well, it is possible to find very marked differences not only between subtypes but within subtypes and mixed infections. Similarly, high diversity has been established in other intestinal microorganisms, mainly in areas of high endemicity, which has been linked with mixed infections that allow recombination and genetic exchange with other microorganisms associated with the accompanying microbiota [43], including *Giardia intestinalis* [44, 45], *Cryptosporidium* sp. [46], and *Entamoeba* [47]. Moreover, the wide variety of hosts and the hostile environmental changes to which these microorganisms are subjected during their transmission facilitates plasticity [48] and genetic variability, as has been observed in trypanosomatid parasites such as *Leishmania* [49] and *Trypanosoma cruzi* [50, 51], or even in other stramenopiles [52]. Similarly, the large number of SNPs found among the genomes subtyped as ST1 with respect to the reference supports the idea of intra-ST variation, as has been reported for assemblages of *Giardia intestinalis* isolates [53].

This is the first study in Colombia and the Americas evaluating genomic data from isolates of *Blastocystis* detected from human samples. It provides evidence of the heterogeneity between ST1 of *Blastocystis*. The new assembly obtained from both Illumina and ONT could be a good reference in future studies where genomic data are necessary to evaluate the architecture of the *Blastocystis* genome and its role at the clinical level, and to elucidate many unknown characteristics of this microorganism. Nevertheless, future studies should determine whether our s20 assembly is adequate or even better than NandII. Ultimately, it will be necessary to establish better assemblies organized by chromosomes to fully investigate variation in the genomic structure of this microorganism for an unprecedented statement to have a good reference genome for *Blastocystis* research.

## Supplementary Information

**Additional file 1**: **Table S1.** Genomic DNAs of Blastocystis isolates used in this study. **Table S2.** Number of SNPs obtained comparing isolated s17 against isolated s20 used as a reference. **Table S3.** Number of SNPs obtained comparing NandII strain against isolated s20 used as a reference. **Table S4.** Number of SNPs obtained comparing isolated s26 against isolated s20 used as a reference. **Table S5.** High-impact SNPs by contig and their product description.

## Data Availability

The dataset generated during the study was deposited at ENA under the bioproject code PRJNA855474.
